# Patent Ductus Arteriosus in Association With Agenesis of Corpus Callosum: Syndromic Links, Difficult Airway Challenges, and Pediatric Anesthesia Management

**DOI:** 10.7759/cureus.63177

**Published:** 2024-06-26

**Authors:** Urvi Sawant, Jayshree Sen, Dhawal Wadaskar

**Affiliations:** 1 Department of Anesthesiology, Jawaharlal Nehru Medical College, Datta Meghe Institute of Higher Education and Research, Wardha, IND

**Keywords:** pediatric cardiac anesthesia, management of complications, pda closure device, patent ductus arteriosis, corpus callosum agenesis

## Abstract

Agenesis of corpus callosum (ACC) is a congenital absence of corpus callosum either completely or partially; without deficits in behavior or function during the first two years of life. Patent ductus arteriosus (PDA) is a congenital cardiac defect in which there is persistent contact between the pulmonary artery and the descending thoracic aorta due to failure of the normal physiologic closure of the fetal ductus. This article details a unique case of a three-month-old male infant who was initially diagnosed with PDA and later discovered to have corpus callosum agenesis. The child was posted on a PDA device for closure. Here, we will be discussing syndromic association, difficult airway, procedure-related factors, and pediatric anesthesia management of this rare case.

## Introduction

The corpus callosum is the biggest white matter structure in the brain, which connects the two hemispheres with 200 million axons [1]. At 74 days gestation, it starts to take shape; at 11 or 12 weeks, the first fibers cross the midline; at about 115 days, development is complete; and at 18 or 20 weeks, the rudimentary shape is reached [2]. Primarily, the genu forms first and the rostrum lasts, forming from anterior to posterior. The complete or partial failure of the development of the corpus callosum causes this congenital neurological anomaly known as Agenesis of corpus callosum (ACC) and is not accompanied by problems in behavior or function (like autism).

Corpus callosum agenesis may manifest as a separate ailment or in conjunction with additional brain anomalies. There exists a link between multiple factors and the etiology of corpus callosum agenesis [3]. Alcohol consumption during pregnancy, maternal phenylketonuria, and Chiari II malformation are some of the etiologic factors [1,4,5]. Twenty percent of ACC cases had chromosomal abnormalities, particularly trisomies 18 and 13. Basal cell nevus syndrome and Apert syndrome (autosomal dominant), Lyon syndrome, Joubert syndrome (autosomal recessive), hydrocephalus or X-linked aqueductal stenosis, and Aicardi syndrome (X-linked) are among the syndromes that have corpus callosum agenesis as an associated characteristic [6].

Failure of the usual physiologic closure of the fetal ductus, which leaves the descending thoracic aorta and pulmonary artery in constant communication results in patent ductus arteriosus (PDA). The ductus arteriosus functions as a fetal conduit enabling oxygenated blood from the placenta to avoid the lungs while the fetus is still inside the body. The first breaths at birth cause the lungs to fill with air, fall in pulmonary vascular resistance, and flow of blood from the right ventricle into the lungs for oxygenation. As a result of increased arterial oxygen tension, the ductus constricts and leads to reduced flow via the ductus arteriosus. When a healthy, full-term newborn is 12 to 24 hours old, the ductus arteriosus is effectively closed. Within two to three weeks, the permanent (anatomic) closure is complete. Pharmacologic or surgical closure to treat side effects may be required in a premature infant, in which the ductus arteriosus does not rapidly close physiologically [7].

## Case presentation

A three-month-old male child, 2.76 kg weight, born from a non-consanguineous marriage, was brought by parents for failure to gain weight for about one month and recurrent respiratory tract infections since birth that were not associated with respiratory distress or cyanosis. The birth weight was 2.2 kg, but the baby did not cry at birth and had a history of Neonatal Intensive Care Unit (NICU) stay for four days. Immunization and developmental history were up to date. After a thorough investigation by the pediatrician, the patient was diagnosed with ACC on MRI brain and PDA using a 2D echo. PDA device closure was planned. The patient was receiving Furosemide drops (2mg/kg/day), Tab. Enalapril (0.1mg/kg/day) and syrup Levetiracetam 0.5 mL twice a day.

**Figure 1:**
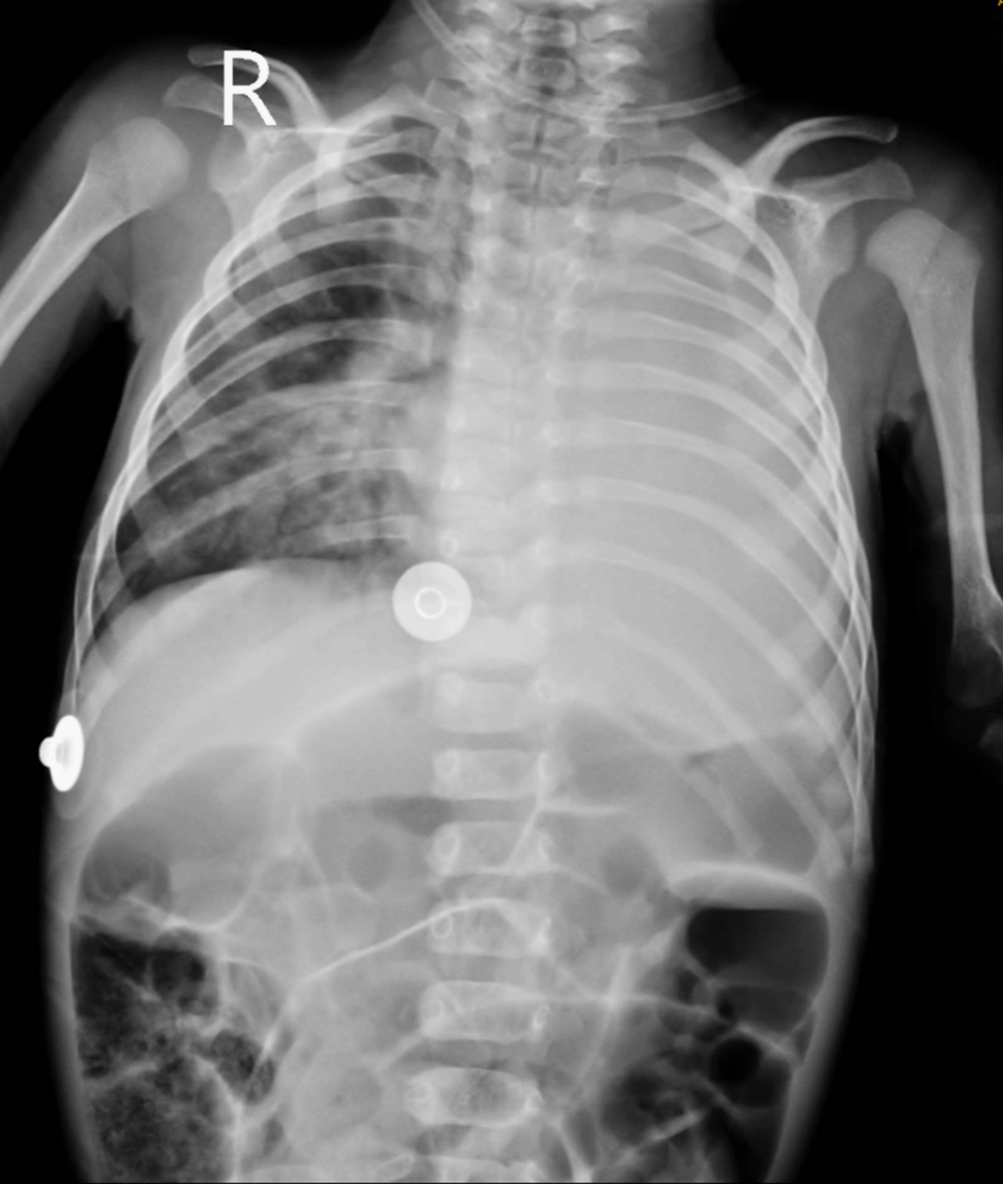
Pre-operative chest radiograph showing diffuse haziness in the left lung field

The patient underwent a meticulous pre-anesthetic evaluation, where on examination, he was found to be vitally stable with a pulse rate of 136/min, blood pressure of 80/48mmHg, respiratory rate of 38/min, and oxygen saturation of 98% on room air. His general condition was moderate, but he had severe malnutrition as per the Indian Academy of Pediatrics grading (weight 2.76 kg, length 40 cm). Anticipated difficult airway was diagnosed by retrognathia and short neck. On inspection, chest retractions were present, and on auscultation, a pan systolic murmur was heard (continuous machinery murmur). All the laboratory blood parameters were within normal limits. MRI of the brain revealed ACC with colpocephaly and an arachnoid cyst in the right retro-cerebellar region. Additional findings noted in the ultrasonography of the abdomen and pelvis were gall bladder calculi suggestive of cholelithiasis, splenomegaly (6.1cm), and cystitis in the urinary bladder. Chest radiography showed diffuse haziness in bilateral lung fields (Figure 1).

**Figure 2:**
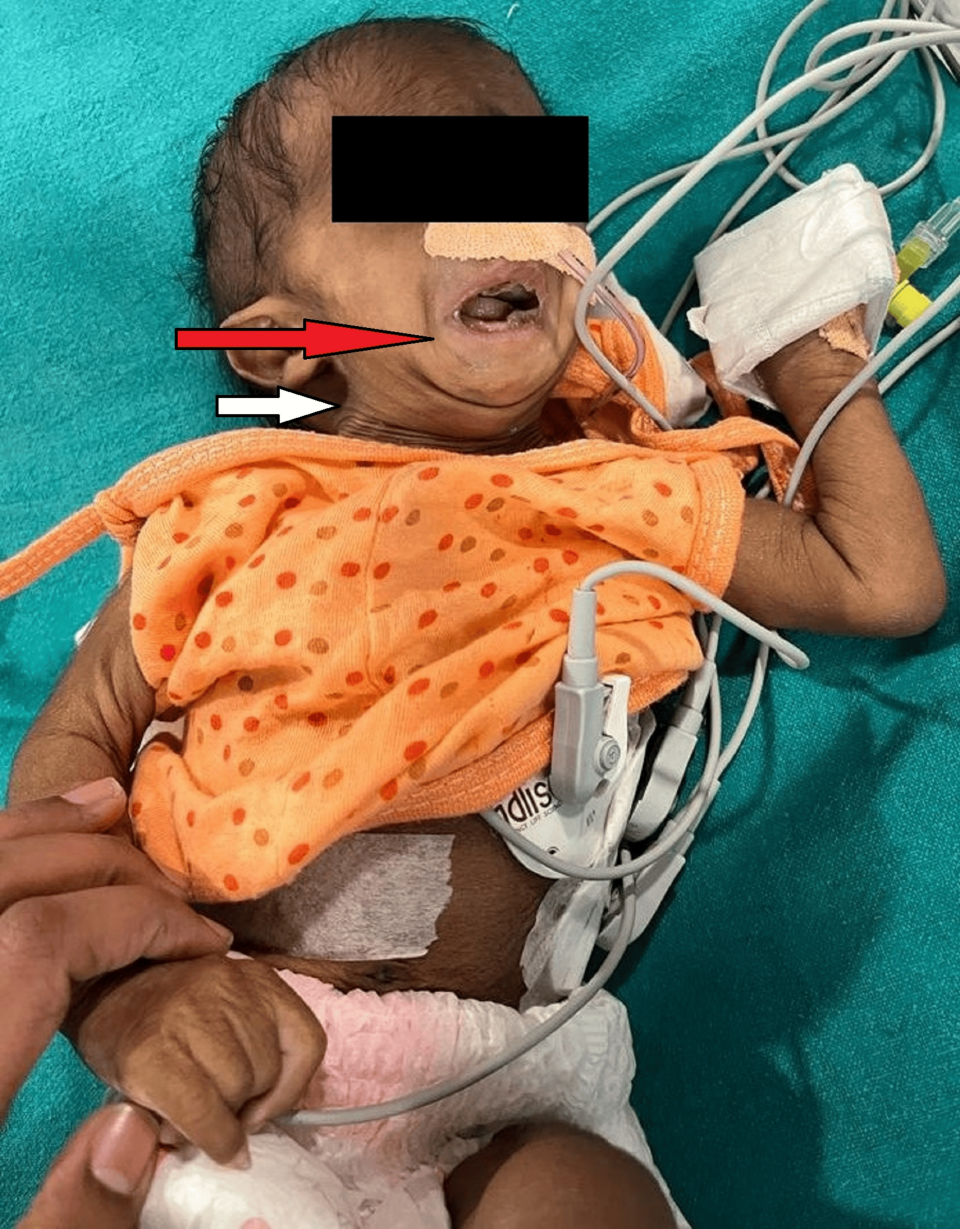
The red arrow shows retrognathia and the white arrow shows a short neck indicative of anticipated difficult intubation

Nil per oral for more than two hours for clear fluid was confirmed on the day of the procedure. After attaching ASA monitors, pre-induction vitals were noted. The patient was euthermic with a pulse of 146/min, blood pressure of 82/54 mmHg, and oxygen saturation of 100% on four liters of oxygen delivered via a Hudson mask. A 24G IV cannula was secured. The patient was covered with a warming blanket to prevent hypothermia. Premedication with Inj. Glycopyrrolate 0.004 mg/kg and Inj. Midazolam 0.02mg/kg were given. Inj. Fentanyl 1 mcg/kg was administered, and anesthesia was induced with inhalation of sevoflurane in incremental doses. The airway was secured using a 3.0 uncuffed endotracheal tube after administering succinylcholine 1-2 mg/kg using direct laryngoscopy. The difficult airway cart was kept ready beforehand. It took three attempts to secure the airway as it was a case of difficult intubation (Figure 2).

As soon as the procedure was over, the patient went into the sudden onset of bradycardia of 73/minute, desaturation up to 85% on room air, and hypotension up to 68/28 mmHg. Cardiopulmonary resuscitation (CPR) as per pediatric Advanced Life Support protocol was started [8]. Inj. Atropine 0.06 mg IV, Inj Adrenaline 25 mcg IV given. After five cycles of CPR, return of spontaneous circulation was achieved, and the patient was resuscitated successfully and shifted to the cardiac intensive care unit on ventilatory support for further management.

**Figure 3:**
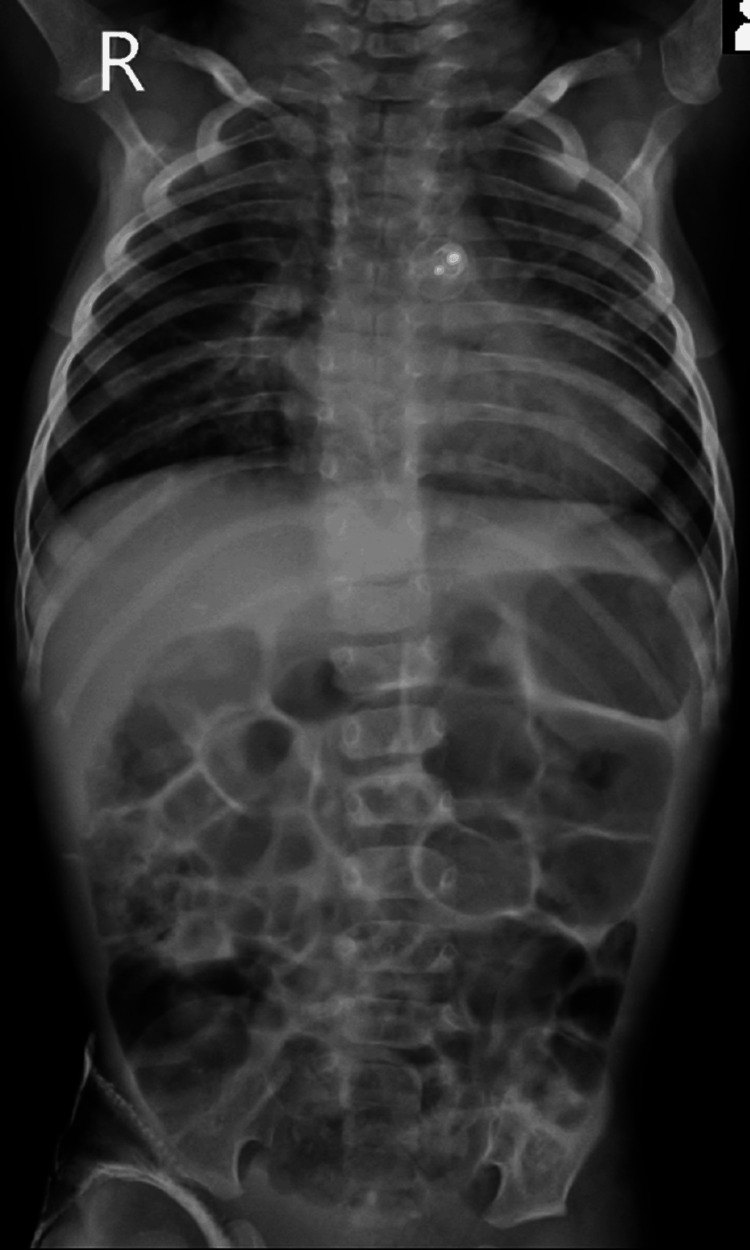
Post-operative chest radiograph showing bilaterally clear lung fields with amplatzer PDA device in situ. Significant improvement was seen symptomatically as well as in the chest radiograph post surgery and mechanical ventilation.

The patient was weaned off the ventilator and extubated the next day successfully, subsequently shifted to the pediatric intensive care unit (PICU), and handed over to a pediatric resident for further management. The patient received a unit of packed red cell transfusion in the PICU postoperatively. Follow-up chest radiography showed bilaterally clear lung fields with the PDA Amplatzer device (Abbott, Green Oaks, IL) in situ. He improved symptomatically and was discharged. The patient is doing well at home as per the last follow-up (Figure 3).

## Discussion

This case highlights the intricate management of a three-month-old male infant with a combination of congenital anomalies (agenesis of the corpus callosum PDA) and other systemic manifestations (cholelithiasis and splenomegaly). Severe malnutrition, failure to thrive, and recurrent respiratory infections were noted on examination. The case highlights the requirement for a multi-disciplinary approach, meticulous peri-operative monitoring, and preparedness to deal with unforeseen and a life-threatening complication of the procedure and clearly shows that favorable outcomes can be achieved in high-risk pediatric groups.

Corpus callosum agenesis and PDA are not known to be directly causally related. However, both conditions are congenital, and they can occur independently in the same patient. In the pediatric emergency room, pediatric intensive care unit, and surgical setting, maintaining the child's airway is an essential part of patient care. Mask ventilation, direct laryngoscopy, and endotracheal intubation techniques are comparatively more challenging in children due to changes in anatomy and physiology. Furthermore, a doctor has less time to complete these tasks for a pediatric patient than an adult. Many of the disastrous results of airway management may be avoidable if thorough assessment, planning, and execution have been done [9].

Following surgical correction of PDA, pulmonary and cardiovascular maladaptation results in a severe low cardiac output, which is known as PLCS, a rare but serious complication. Because of the ductus arteriosus closing, PLCS is associated with an acute increase in afterload and a decrease in preload. In a PDA condition, the left atrium is overloaded due to high pulmonary flow, and the pulmonary vascular bed provides low resistance to the left ventricle (low afterload) (high preload). The left ventricle, however, experienced an abrupt increase in afterload (no longer a low-resistant pulmonary vascular bed) and a decrease in preload as soon as the ductus was closed. Therefore, there is a chance of both systolic and diastolic dysfunction, which lowers cardiac output [10].

Neurological assessment both before and after the procedure is a must because the corpus callosum contributes to interhemispheric communication [11]. It is important to record any prior neurological impairments and assess baseline cognitive performance. Monitoring any changes in the nervous system both during and after the procedure can be aided by this. Patients who have corpus callosum agenesis are more likely to experience seizures which should be taken into account in the anesthetic plan, and prophylactic measures or appropriate antiepileptic drugs may be required to limit the risk of seizures.

Exercise caution when positioning patients to avoid aggravating any musculoskeletal problems that may be related to their disease. Take into account the possible effects on any underlying neurological deficiencies while selecting anesthetic medications. Intraoperative Monitoring is essential to keep a close eye on key indicators such as blood pressure, oxygen saturation, and heart rate. In order to evaluate cerebral perfusion and oxygenation, we should also think about using cerebral oximetry or other neuromonitoring methods. Close observation is necessary during the recovery phase, especially if the patient has a history of seizures or other neurological problems. The postoperative treatment plan should include adequate pain control as well as prompt intervention in the event of any neurological impairment.

## Conclusions

Drug dosages for pediatric anesthesia patients must be calculated individually based on their weight and age. Infants and children are at risk of hypothermia, difficulty in IV access, difficult airway, risk of fluid overload, disease-related factors, and procedure-related factors. Syndromic associations can furthermore cause complications intraoperatively. An interdisciplinary team approach is essential for arranging the anesthetic management of a patient undergoing PDA device closure who has corpus callosum agenesis. This includes consulting with a neurologist or pediatric neurologist. A safe and effective procedure can be ensured by addressing any neurological concerns and customizing the anesthesia to the patient's specific needs. A satisfactory post-operative result and proper management are facilitated by early forecasting of problems and the prognosis. The previously mentioned issues were taken into account and handled carefully in this novel case.
